# Statistical Analysis of Reproductive Traits in Jinwu Pig and Identification of Genome-Wide Association Loci

**DOI:** 10.3390/genes16050550

**Published:** 2025-04-30

**Authors:** Wenduo Chen, Ayong Zhao, Jianzhi Pan, Kai Tan, Zhiwei Zhu, Liang Zhang, Fuxian Yu, Renhu Liu, Liepeng Zhong, Jing Huang

**Affiliations:** 1 College of Animal Science and Technology & College of Veterinary Medicine, Zhejiang A&F University, Hangzhou 311300, China; 19550178717@163.com (W.C.); zay503@zafu.edu.cn (A.Z.); 2 Institute of Virology and Biotechnology, Zhejiang Academy of Agriculture Science, Hangzhou 310021, China; jzpan9@126.com (J.P.);; 3 College of Animal Sciences, Fujian Agriculture and Forestry University, Fuzhou 350002, China

**Keywords:** reproductive traits, genome-wide association analysis, Jinwu pig, SNPs

## Abstract

Background: The Jinwu pig is a novel breed created by crossbreeding Jinhua and Duroc pigs, displaying superior meat quality, strong adaptability to coarse feed, high production performance, and a rapid growth rate. However, research on its reproductive traits and genomic characteristics has not been systematically reported. Methods: In this study, we investigated the genetic basis of reproductive traits in Jinwu pigs us-ing a genome-wide association study. We analyzed 2831 breeding records from 516 Jinwu sows to evaluate the effects of fixed factors (farrowing season, parity, and mated boar) on six reproductive traits: the total number of births (TNB), number born alive (NBA), number of healthy offspring produced (NHOP), weak litter size (WLS), number of stillbirths (NS), and number of mummies (NM). Results: A total of 771 genome-wide significant single-nucleotide polymorphisms (SNPs) and ten potential candidate genes associated with pig reproductive traits were identified: *VOPP1, PGAM2, TNS3, LRFN5, ORC1, CC2D1B, ZFYYE9, TUT4, DCN*, and *FEZF1*. TT-genotype-carrier individuals of the pleiotropic SNP rs326174997 exhibited significantly higher TNB, NBA, and NHOP trait-related phenotypic values. Conclusions: These findings provide a foundation for the reproductive breeding of Jinwu pigs and offer new insights into molecular genetic breeding in pigs.

## 1. Introduction

Chinese indigenous pig breeds are recognized for their excellent reproductive performance, high-quality meat, and strong resilience to stress [1,2]. However, compared to commercial pig breeds, such as Duroc, Chinese indigenous pig breeds exhibit inferior growth rates, feed efficiency, lean meat percentage, and body size [3,4]. Over ten years have been dedicated to developing the Jinwu pig, a new breed, to balance meat quality and production performance, using traditional crossbreeding and modern molecular breeding techniques. Jinwu pigs were developed by crossing Jinhua pigs, one of China’s four major premium pig breeds, with Duroc pigs. They comprise 37.5% Jinhua and 62.5% Duroc pig lineages, combining the excellent meat quality and adaptability to coarse feed of Jinhua pigs with the rapid growth and high meat production performance of Duroc pigs.

Reproductive performance is a key factor in evaluating pig breeds’ productivity and economic efficiency. However, reproductive traits are typically characterized by low heritability (~0.1) [5] and complex genetic structures, limiting genetic improvement in sow reproductive performance through traditional breeding methods. With advancements in molecular biology, marker-assisted selection (MAS) and genomic selection (GS) have been increasingly adopted [6], significantly enhancing breeding efficiency.

Genome-wide association studies (GWASs) have recently emerged as a powerful tool for investigating the genetic basis of complex traits and identifying quantitative trait loci (QTLs) associated with economically important traits. The most recent version of PigQTLdb (23 December 2024) reported 56,615 QTLs in pigs (https://www.animalgenome.org/cgi-bin/QTLdb/SS/summary), accessed on 17 February 2025. among which 7338 are related to reproductive traits. Numerous SNPs and candidate genes related to economically important traits in pigs have been successfully identified, including those related to reproduction [7,8,9], growth [10,11,12], and meat quality [13,14,15]. These data provide a wealth of molecular markers for pig breeding.

Jinwu pigs, crossbreeds of Chinese native pigs and commercial breeds, have not had their reproductive performance systematically documented or analyzed, and the genetic factors influencing their reproductive traits have been partially characterized. Improving reproductive performance is crucial for enhancing the production efficiency of Jinwu pigs, with considerable implications for their genetic improvement and industrialization.

This study systematically analyzes the reproductive traits of Jinwu pigs, including the total number of births (TNB), number born alive (NBA), number of healthy offspring produced (NHOP), weak litter size (WLS), number of stillbirths (NS), and number of mummies (NM), as well as the influence of fixed effects on these traits. Moreover, by integrating whole-genome resequencing data, modern molecular breeding methods, including a GWAS, are employed to identify SNPs, QTL regions, and candidate genes associated with reproductive traits. This study offers insights into improving Jinwu pigs’ reproductive performance and provides new perspectives on the genetic analysis of reproductive traits in pigs.

## 2. Materials and Methods

### 2.1. Laboratory Animals and Phenotypic Statistical Analysis

Reproductive records of Jinwu pigs were collected from Zhejiang Huateng Animal Husbandry Co, Ltd., Anji, Zhejiang, China, spanning from 2022 to 2024. The phenotypic records included parity (1 to 7 or higher), season, and the mated boar. Six reproductive traits were selected for analysis: TNB, NBA, NHOP, WLS, NS, and NM. Based on data distribution and previous studies, records with TNB, NBA, or NHOP values <3 or >30 were excluded [16]. After data cleaning and organization, 516 Jinwu sows and 2831 reproductive records were collected. Table 1 presents the phenotypic statistical descriptions of the reproductive traits.

### 2.2. Genotyping and Quality Control

The kit used for sample extraction was the Magnetic Universal Genomic DNA Kit, provided by Beijing Novogene Technology Co., Ltd., Beijing, China, using a magnetic bead-based extraction method. First, a sample from the animal ear tissue was collected, placed into a 1.5 mL EP tube, and finely cut into pieces. The sample was mixed with the lysis buffer, allowing DNA to bind to magnetic beads, followed by a series of washing steps. Finally, the DNA was eluted from the magnetic beads to obtain high-quality genomic DNA. For specific operations, refer to the DP705 kit instructions. Low-coverage whole-genome sequencing was performed on 516 samples using the DNBSEQ-T7 platform, with an average sequencing depth of 2.17×. Raw sequencing reads were filtered using fastp v0.20.0 [17] under default parameters to remove low-quality reads. Clean reads were processed using GTX v2.1.5 [18] followed by genotype evaluation and the filling of low-depth sequencing data using GLIMPSE2 software v2.0.0 [19]. Next, SNPs with INFO_scores < 0.4 were filtered using bcftools v1.12. Finally, PLINK v1.9 [20] was used under “--geno 0.1 -maf 0.05 -mind 0.1 -hwe 1 × 10^−10^” to ensure the reliability of the data. After rigorous screening, 14,525,958 high-quality SNPs were obtained.

### 2.3. Fixed Effects’ Statistical Modeling

A multifactorial analysis of variance (ANOVA) and significance tests were performed to analyze the effects of three fixed factors (boar, season, parity) on six reproductive traits using the aov function of the R software (version 4.4.1).

The specific model is represented by Equation (1):

(1) $$Y_{ijkl}=\mu +B_{i}+S_{j}+P_{k}+e_{ijkl}$$

where $Y_{ijkl}$ is the observed value of each trait; μ is the population mean; $B_{i}$ is the boar effect; $S_{j}$ is the seasonal effect at four levels: spring (March–May), summer (June–August), fall (September–November), and winter (December–February); $P_{k}$ is the parity effect for 1–7 parities; and $e_{ijkl}$ is the random residual effect.

### 2.4. Estimation of Genetic Parameters

The heritability and variance components of the reproductive traits were estimated using the Average Information Restricted Maximum Likelihood (AI-REML) method in the HIBLUP [21] software (v1.5.3).

The fitted single-trait model is represented by Equation (2):

(2) y=Xb+Zu+e

where y is the phenotypic value, β is a fixed effect (parity, mated boars, season), *u* is a random additive genetic effect where the genomic relationship G matrix was constructed based on the VanRaden [22] method, and *e* is a vector of random residual effects.

### 2.5. Principal Component Analysis

Principal component analysis (PCA) was performed on the imputed genotype data using PLINK v1.9. Since the Jinwu pig is a cross between the Duroc and Jinhua pigs, the Duroc (*n* = 335) and Jinhua (*n* = 161) data were first downloaded from PHARPV4 public data. Subsequently, population structure and genetic diversity analyses, including PCA, neighbor-joining (NJ) tree construction, ADMIXTURE analysis, F_st_ (fixation index), Pi (nucleotide diversity), H_e_ (expected heterozygosity), H_o_ (observed heterozygosity), PIC (polymorphism information content), SHI (population genetic structure index), and purity testing, were conducted across the three pig populations (Jinwu, Duroc, and Jinhua). The top five principal components were visualized using the “ggplot2” package in R.

### 2.6. Genome-Wide Association Study (GWAS)

Genome-wide association analysis was performed using a Mixed Linear Model (MLM) for single traits using GEMMA v0.98 [23] software with Equation (3):

(3) Y=Xβ+Sα+Zu+e

where Y is a vector for the six breeding trait phenotypes of Jinwu pigs (TNB, NBA, NHOP, WLS, NS, NM); β is a covariate (season, mated boar) and the first five principal components; α is the effect of an SNP; u is the microeffective polygenic effect that obeys a multivariate normal distribution of u~N(0, *G*$\sigma_{a}^{2}$), where G is the relationship matrix constructed based on the SNP markers and $\sigma_{a}^{2}$ is the microefficient multigene variance; e is the residual vector with the distribution N(0, I$\sigma_{e}^{2}$), where Ⅰ is the unit matrix; and *X*, *S*, and *Z* are the correlation matrices or vectors of β, α, and u, respectively. *p* < 1 × 10^−6^ was set as the genome-wide level significance threshold. The GWAS analysis was visualized using the CMplot package [24] in R version 4.5.1, and the generated Manhattan and QQ plots showed significant correlations between SNPs and reproductive traits.

### 2.7. Linkage Disequilibrium and Haplotype Analysis

Haplotype regions were constructed and analyzed using Haploview software v4.2 [25] to assess associations with porcine reproductive traits. Focus was placed on the high-intensity linkage disequilibrium of haplotypes and their significance within candidate regions to identify potential candidate genes associated with significant SNPs.

### 2.8. Candidate Gene Annotation

GTF gene annotation files corresponding to the porcine reference genome Sscrofa11.1 were downloaded from the Ensembl database (https://asia.ensembl.org/). Significant SNPs were annotated using VEP [26] to identify their potential functional variants. Subsequently, 0.5 Mb upstream and downstream of the candidate SNPs was annotated in combination with R scripts and the R package GALLO [27] to screen their corresponding genes.

## 3. Results

### 3.1. Phenotypic and Genetic Correlations

**Figure 1:**
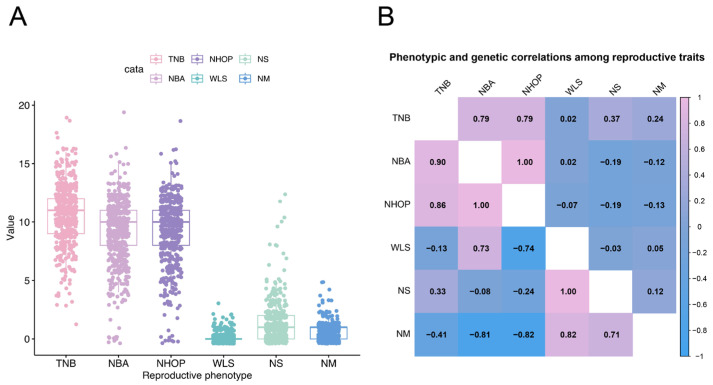
Phenotypic and genetic correlations between reproductive traits in experimental populations. (**A**) Box line plot. (**B**) The upper triangle represents the phenotypic correlations between traits, while the lower triangle represents the genetic correlations.

The distribution and correlations of six reproductive traits—the TNB, NBA, NHOP, WLS, NS, and NM—were analyzed in the Jinwu pig population. The TNB, NBA, and NHOP exhibited a wide range of values, indicating significant individual variability (Figure 1A). This suggested substantial potential for further genetic improvement in the reproductive performance of Jinwu pigs. Figure 1B presents the phenotypic and genetic correlation results. The TNB and NBA displayed the strongest correlation (correlation coefficient of 0.99), indicating a high degree of consistency between these two traits. In contrast, the NS and NM exhibited negative correlations with the NBA and NHOP (correlation coefficients ranging from −0.15 to −0.19). The genetic correlation matrix showed genetic associations among different reproductive traits. The genetic correlation coefficients of the TNB with the NBA and NHOP were 0.90 and 0.86, respectively, showing a strong positive correlation.

### 3.2. Factors Affecting the Reproductive Performance of Jinwu Sows

The effects of fixed factors on the reproductive traits of Jinwu sows are summarized in (Table 2). Mating boar had a significant effect (*p* < 0.05) on all traits except TNB and NS. Season significantly influenced all traits except NS (*p* < 0.001), while parity exhib-ited a very highly significant influence on all reproductive traits (*p* < 0.001).

Season had significant effect on reproductive traits of Jinwu sows (Table 3). Sum-mer and fall were optimal in NBA (10.30 and 10.28 head, respectively) and NHOP (10.27 and 10.28 head, respectively), lower in WLS (0.03 and 0 head, respectively), and differed significantly in reproductive traits in summer and fall compared to winter (*p* < 0.05); TNB (11.34 head) was significantly lower in winter than spring and summer; Although spring had the highest TNB (11.78), it was also associated with significantly higher NS and NM compared to other seasons.

Parity significantly influenced the reproductive traits of Jinwu sows (Table 4). TNB, NBA, and NHOP gradually increased with parity, peaking at the sixth parity (12.18 ± 0.13 and 10.42 ± 0.11 piglets per litter). A slight decline was observed at the seventh parity (12.16 ± 0.15, 10.34 ± 0.13, and 10.3 ± 0.13 piglets per litter), although reproduc-tive performance remained relatively high. In contrast, no significant differences were observed in NBA or NHOP across different parities.

### 3.3. Estimation of Genetic Parameters for Reproductive Traits in Pigs

The genetic parameters of the six reproductive traits are shown in Table 5. The heritability of the TNB, NBA, NHOP, WLS, NS, and NM was 0.0894 ± 0.0256, 0.0918 ± 0.0240, 0.0895 ± 0.0237, 0.0085 ± 0.0082, 0.0029 ± 0.0068, and 0.0136 ± 0.0102, respectively. Thus, the heritability of reproductive traits in the experimental population was consistently <0.1.

### 3.4. Principal Component Analysis

**Figure 2:**
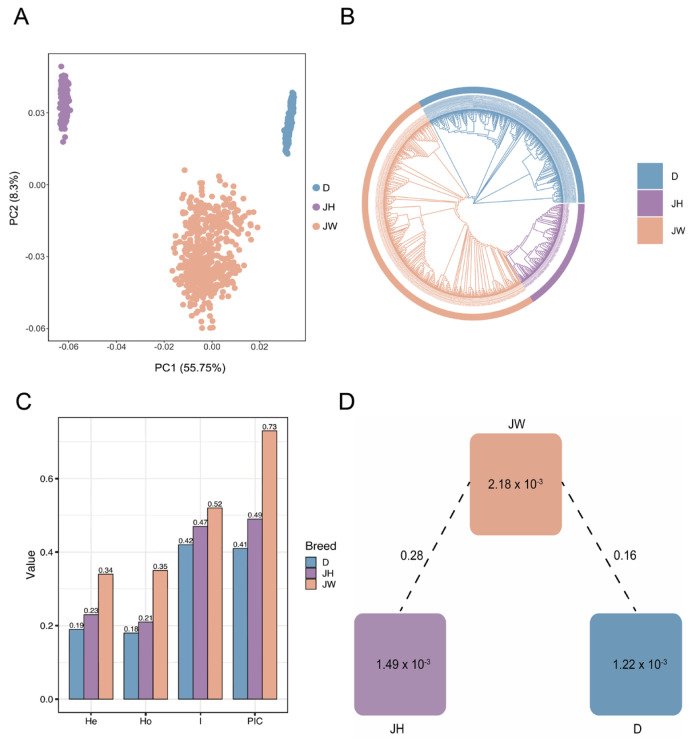
Population genetic analysis. (**A**) Principal component analysis (PCA) results for three pig varieties. (**B**) Phylogenetic tree diagram. (**C**) Genetic diversity indices. (**D**) Genetic differentiation plot of within−population heterozygosity ratios. Note: D: Duroc; JH: Jinhua pig; JW: Jinwu pig.

The differences in the population structure of the three varieties Duroc, Jinhua, and Jinwu were analyzed by PCA; Jinwu was significantly segregated from the other varieties in PC1 and PC2, demonstrating its unique genetic structure (Figure 2A). The phylogenetic tree was consistent with the PCA results and successfully divided into three populations showing genetically distinct clusters (Figure 2B). The Jinwu variety exhibited a higher H_e_, H_o_, and PIC than Duroc or Jinhua, indicating rich genetic diversity (Figure 2C). The F_st_ value reflects the degree of genetic differentiation between populations, with higher values indicating greater genetic differences between populations [28] (Figure 2D). The F_st_ value for Jinwu and Jinhua (0.28) was significantly higher than that for Jinwu and Duroc (0.16), indicating a greater genetic distinction between Jinwu and Jinhua. This further suggested that Jinwu has genetically differentiated during the artificial selection and breeding process, gradually forming independent genetic characteristics.

### 3.5. Genome-Wide Association Study

**Figure 3:**
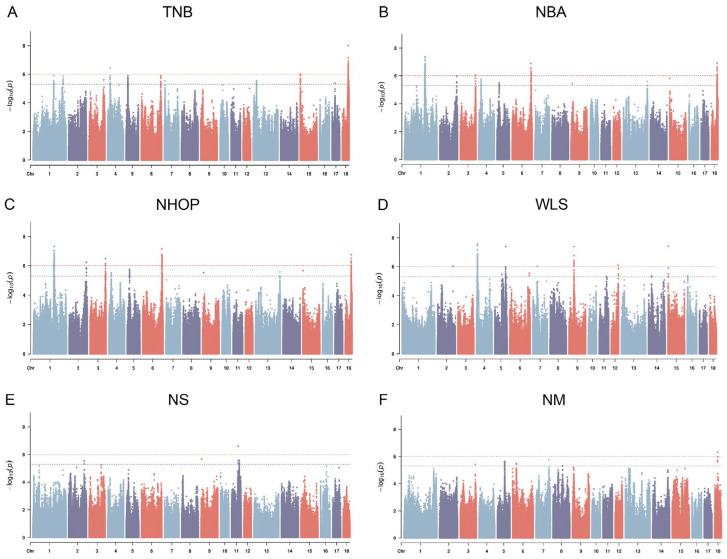
Manhattan plot of GWAS results for reproductive traits: (**A**) total number of births (TNB), (**B**) number born alive (NBA), (**C**) number of healthy offspring produced (NHOP), (**D**) weak litter size (WLS), (**E**) number of stillbirths (NS), and (**F**) number of mummies (NM); *Y*-axis: −log10 of *p*-values; *X*-axis: chromosome number; red dashed line: threshold line for significant loci at the whole-genome level (*p* < 1 × 10^−6^); blue dotted line: potential significant threshold.

The Manhattan plots of the GWAS analysis are shown in Figure 3A–F, and the QQ plots are shown in Figure S1a–f. The results identified a total of 771 significant SNP loci (*p* < 1 × 10^−6^), distributed in multiple chromosomal regions (Table S1). The vep results for the TNB, NBA, and NHOP showed that 69% of the loci were intronic variants, 20% were intergenic regions, and the remainder were distributed in downstream gene regions (5%), noncoding transcript regions (2%), regulatory regions (1%), etc. Most significant SNP loci were located in noncoding regions (Table S1). Subsequent analysis of the GWAS significant loci focused on significant SNPs (pleiotropic SNPs) that recurred in multiple traits (TNB, NBA, NHOP). As shown in Table 6, these significant SNP loci are closely associated with multiple traits. In addition, regarding the distribution of candidate genotypes for pleiotropic SNPs, the relative frequencies of the relevant genotypes in different breeds (Jinwu, Duroc, and Jinhua pigs) are shown in Supplementary Table S3.

**Figure 4:**
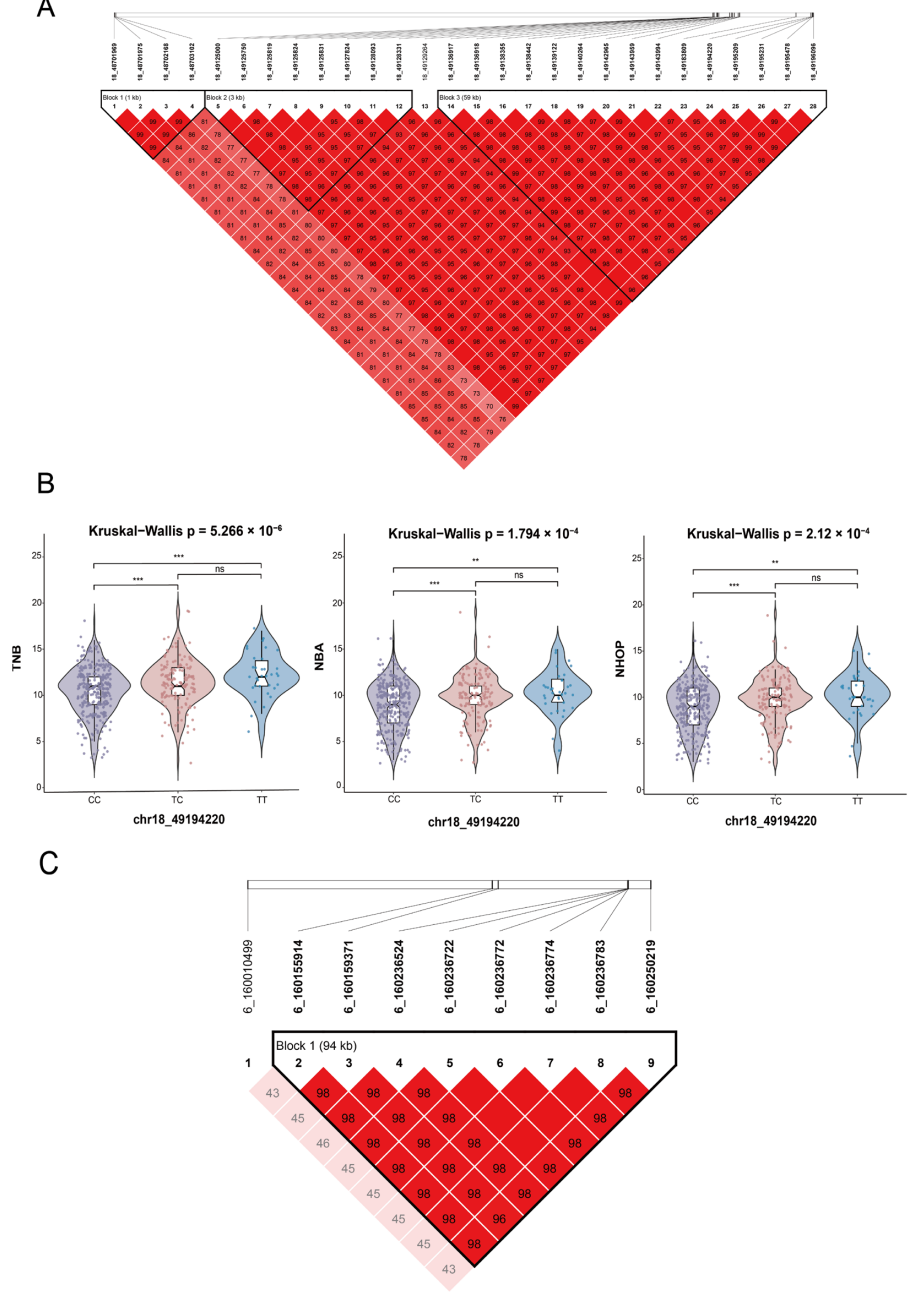
Regional linkage disequilibrium (LD) analysis results. (**A**) Haplotype blocks formed on SSC18 by the significant SNP chr18_49194220 associated with the TNB, NBA, and NHOP. (**B**) Genotype effect plot of the pleiotropic SNP chr18_49194220 showing its effect on the TNB, NBA, and NHOP, with overall intergroup differences analyzed using the Kruskal–Wallis test; differences between two groups were analyzed using the Wilcoxon test, with significance levels denoted by ** (*p* < 0.01) and *** (*p* < 0.001); ns: no significant difference. (**C**) Distribution of significant loci associated with the NBA and NHOP in the SSC6 chromosomal region and their linkage disequilibrium (LD). Note: In all plots, the colors represent the degree of linkage disequilibrium (LD): **red** indicates high LD (e.g., >95%), and lighter colors represent lower LD values.

For SSC18, chr18_49194220 (rs326174997, C > T) was significant in all three traits, the TNB, NBA, and NHOP (Table 6). According to the vep annotation, this locus is located in the downstream region of the candidate gene *TNS3*. Effect value analysis showed that individuals with the TT genotype exhibited higher phenotypic values in all three traits (Figure 4B). To explore the genetic background of this locus, haplotype analysis of the 49.12–49.27 Mb region of SSC18 was performed (Figure 4A). Three LD blocks were formed in this region, while the remaining SNPs were highly interlinked with rs326174997 (r^2^ > 0.8). Other notable SNPs within the region were annotated to multiple genes (*VOPP1*, *PGAM2*, *DBNL*, *UBE2D4*, *C7orf57*, *FAM221A*, *UPP1;* Table S2). In summary, rs326174997 is a significant genetic marker, and the significance of the region in which it is located suggests that it may be expressed in *TNS3* and neighboring genes.

In the context of the NBA and NHOP, pleiotropic SNPs were identified in chromosomes 1 and 6 (Table 6). According to the vep annotation, the most significant locus of SSC1, 1_172136167 (rs80793150, T > C), was in the intron of the candidate gene *LRFN5*. On chromosome 6, the significant locus 6_160159371 (rs332416322, G > A) was in the intron of the candidate gene *TXNDC12*; however, no clear association was detected between this gene and reproductive traits. The 0.5 Mb region upstream of this SNP was annotated to *ORC1*, *CC2D1B*, *ZFYVE9*, and *TUT4* to further explore potential genes associated with the traits. Preliminary analysis determined that these genes might be related to reproductive traits. Moreover, the pleiotropic SNP 6_160159371 with the neighboring SNP locus (6:160155914–160250219 bp) constituted a 94 kb haplotype block (Figure 4C), with loci showing a high degree of interlocking (r^2^ > 0.8).

Finally, six SNPs were identified in chromosomes 2, 4, 5, 9, 12, and 15 associated with WLS, namely 2_129873417, 4_7866356, 5_91683774, 9_41270949, 12_56294122, and 15_2585414, localized to *MARCHF3*, *LMNB1 ST3GAL1*, *DCN*, *ENSSSCG00000015049*, *DNAH9*, and *LYPD6,* respectively. Additionally, SNP18_24846807 was identified on chromosome 18 associated with the NM; the nearest genes were *FEZF1* and *CADPS2*. However, no related genes were associated with the NS (Table S2).

## 4. Discussion

We conducted the first systematic phenotyping of reproductive traits in Jinwu pigs. The GWAS results revealed several significant SNPs repeatedly associated with traits such as TNB, NBA, and NHOP, suggesting that these traits may share a common ge-netic basis. This association was further supported by haplotype analysis. In addition, all six reproductive traits exhibited low heritability (ranging from 0.0085 to 0.0918), indicating that their variation is largely influenced by environmental and management factors. These findings are consistent with previous studies [16,29]. In addition, we calculated the genetic correlations between individual traits and found high genetic correlations between the TNB, NBA, and NHOP at 0.86–0.90, which is similar to the results of previous studies [30,31].

In the phenotypic data analysis, the season and parity were identified as factors that significantly influence the reproductive traits of Jinwu pigs. Parity analysis revealed that the TNB and NBA gradually increased with parity. Unlike findings in other studies [32,33], Jinwu pigs maintained a high TNB at the sixth and seventh parities. Additionally, in our statistical analysis, we found no significant difference in litter size between the first and second and subsequent parities of Jinwu pigs, which was different from previous studies [34,35], This may be related to the genetic background of the Jinwu breed, which includes a large number of local Jinhua pig breeds. Sows of this breed tend to reach sexual maturity early, usually around 15 days old, and the first mating usually occurs between 7.5 and 8.5 months of age, which corresponds to ap-proximately the fifth estrous cycle. The number of ovulations at the time of the first mating is already close to that of mature sows, which is different from the practice of hybrid sows that usually mate for the first time in the second estrous cycle. Therefore, the reproductive performance of Jinwu pigs is close to the mature level at the time of the first mating, resulting in no significant difference in litter size between the first and subsequent parities. Based on this characteristic, it is recommended that in future production practices, the breeding time of Jinwu pigs can be appropriately advanced to further optimize their reproductive performance.. The effect of the farrowing season on reproductive traits is primarily due to heat stress caused by environmental changes. The winter and spring enhance sows’ reproductive performance, whereas the summer typically results in the poorest outcomes [36]. However, in the current study, the NM and NS in Jinwu pigs were not influenced by the season. Although the highest TNB was observed in the spring, the increased litter size may lead to greater competition, resulting in lower live birth rates and higher stillbirth rates. The NBA was highest in the summer, while reproductive performance was lowest in the winter. Variations in research findings across studies may be attributed to multiple factors, including but not limited to differences in geographical location, housing types, and temperature control equipment, as well as variations in modern management practices [37,38].

For TNB traits, *VOPP1* plays an important role in placental protection and preterm labor mechanisms by regulating oxidative stress and mitochondrial function. Its high expression during pregnancy may reflect the adaptive response of the maternal–fetal interface to environmental stresses and provides a potential molecular marker for preterm labor prediction [39]. *PGAM2* is among the core enzyme-influenced genes of the glycolytic pathway, providing energy for the rapid development of muscle tissues and cellular metabolism, which is essential for organism growth and development [40]. *PGAM2* is functionally conserved in different species, which may indirectly affect reproductive traits, such as embryonic development and growth performance, through metabolic regulation. *TNS3* is not directly related to reproductive traits but helps regulate cell adhesion, skeletal development, and signaling functions [41,42], which provides ideas for follow-up studies to explore the potential role of TNS3 in reproduction.

*LRFN5* was identified at a significant SNP (1_172136167) in SSC1 and found to be associated with the NBA and NHOP. This gene was reported to be up-regulated in ovarian tissue in an analysis of candidate genes identified for traits related to egg production [43]. The closest gene to it, *BTF3L4*, was identified near the significant SNP (6_160159371) in SSC6. However, no study has demonstrated a clear association between *BTF3L4* and reproductive traits in pigs. To further explore possible candidate genes, we expanded the upstream and downstream searches of the significant loci and identified *ORC1*, *CC2D1B*, *ZFYYE9*, and *TUT4* in association with the NBA and NHOP. *ORC1* is involved in ciliogenesis and is key in DNA replication; cilium function is critical for mammalian oocyte transport and embryo development [44]. Meanwhile, *CC2D1B* regulates follicular development and maturation in the ovarian microenvironment, and deletion or mutation may lead to ovarian dysfunction. This gene is also closely related to sperm head formation and sperm function [45]. The *ZFYVE9* gene is considered a candidate gene closely related to ovulation rate and litter size by affecting protein expression related to the TGF-β signaling pathway (SMAD2 and 3), which in turn affects ovulation, follicle development, and other reproductive processes [46]. *TUT4* contributes to oocyte maturation, post-fertilization mRNA remodeling, and early embryonic development by regulating the uridylation and degradation of maternal mRNA. A lack of *TUT4* disrupts mRNA metabolism, affecting the first cleavage of the embryo and ultimately leading to reproductive disorders [47]. In the context of WLS, *DCN* regulates nutrient supply between the mother and fetus as well as fetal growth by contributing to the development of placental structures and collagen fiber formation. The abnormal expression of *DCN* may affect placental function, leading to fetal growth retardation or weak fetuses [48]. *FEZF1* was found to be associated with the NM [49], which contributes to nervous system development, particularly the olfactory system and neuroendocrine function. Therefore, *FEZF1* may indirectly affect the reproductive behavior and reproductive cycle of animals by regulating olfactory perception and the neuroendocrine system.

However, in the current study, no genes were identified as being related to the NS, likely due to the small population size and the numerous missing phenotypic data points. Nevertheless, this study provides valuable insights into the genetic basis of reproductive traits in Jinwu sows. In the future, RNA-seq could be used to analyze candidate gene expression in reproductive tissues, ChIP-seq could be applied to study transcription factor binding and histone modifications, and mouse knockout experiments could prove useful in validating the functions of these genes, which would further improve the current study and promote the progress of the GWAS analysis of porcine reproductive traits.

## 5. Conclusions

This study focused on the phenotypic analysis and genetic parameter estimation of reproductive traits (TNB, NBA, NHOP, WLS, NS, NM) in Jinwu pigs, which are characterized by low heritability. Through a GWAS, 771 SNPs associated with reproductive traits were identified. After reviewing the literature, ten potential candidate genes were selected: *VOPP1, PGAM2, TNS3, LRFN5, ORC1, CC2D1B, ZFYYE9, TUT4, DCN,* and *FEZF1*. Several of these genes have not been previously examined in livestock but might serve as crucial candidates influencing reproductive traits in pigs. This study contributes to elucidating the genetic architecture of reproductive traits in Jinwu pigs and offers a promising basis for future genetic improvement in pig breeding programs.

## Figures and Tables

**Table 1:** Summary statistics for six reproductive traits.

Trait ^1^	Litters ^2^	CV (%) ^3^	Mean	SD	Max	Min
TNB	2831	21.49	11.65	2.5	24	3
NBA	2809	21.49	10.15	2.18	19	3
NHOP	2808	21.58	10.12	2.18	19	3
WLS	2831	709.56	0.03	0.21	3	0
NS	2831	119.16	1.08	1.29	12	0
NM	2831	152.68	0.48	0.74	7	0

^1^ TNB: total number born; NBA: number born alive; NHOP: number of healthy offspring produced; WLS: weak litter size; NS: number of stillbirths; NM: number of mummies. ^2^ Litters: litter size. ^3^ CV: coefficient of variation.

**Table 2:** Significance test for fixed effects of reproductive traits in Jinwu sows.

Trait ^1^	Boar ^2^		Season		Parity	
	df	F	df	F	df	F
TNB	40	1.39	3	4 **	6	14.14 ***
NBA	40	2.06 ***	3	3.81 **	6	13.87 ***
NHOP	40	2.23 ***	3	3.88 **	6	16.1 ***
WLS	40	1.42 *	3	11.09 ***	6	11.48 ***
NS	40	1.19	3	22.84 ***	6	7.38 ***
NM	40	1.45 *	3	3.19 *	6	7.05 ***

Note: ^1^ TNB: total number born; NBA: number born alive; NHOP: number of healthy offspring produced; WLS: weak litter size; NS: number of stillbirths; NM: number of mummies. ^2^ Boar: refers to the male pigs used for breeding purposes. * indicates significant, *p* < 0.05; ** indicates highly significant, *p* < 0.01; *** indicates extremely significant, *p* < 0.001.

**Table 3:** Seasonal effects on reproductive traits.

Trait ^1^	Spring	Summer	Autumn	Winter
TNB	11.78 ± 2.6 ^a^	11.76 ± 2.41 ^a^	11.69 ± 2.46 ^ab^	11.34 ± 2.52 ^b^
NBA	9.8 ± 2.52 ^a^	10.3 ± 2.13 ^a^	10.28 ± 2.24 ^b^	9.92 ± 2.38 ^b^
NHOP	9.77 ± 2.52 ^a^	10.27 ± 2.12 ^a^	10.28 ± 2.25 ^b^	9.84 ± 2.4 ^b^
WLS	0.02 ± 0.18 ^a^	0.03 ± 0.25 ^b^	0 ± 0 ^bc^	0.07 ± 0.3 ^c^
NS	1.4 ± 1.59 ^a^	1.01 ± 1.16 ^b^	0.93 ± 1.11 ^b^	0.96 ± 1.17 ^b^
NM	0.56 ± 0.76 ^a^	0.45 ± 0.78 ^ab^	0.46 ± 0.78 ^b^	0.45 ± 0.6 ^b^

Note: ^1^ TNB: total number born; NBA: number born alive; NHOP: number of healthy offspring produced; WLS: weak litter size; NS: number of stillbirths; NM: number of mummies. Different letters in the same row indicate significant differences (*p* < 0.05), while the same letters indicate no significant differences (*p* > 0.05).

**Table 4:** Effects of parity on reproductive traits.

	Parity						
Trait ^1^ Litters ^2^	1st 516	2nd 494	3rd 442	4th 357	5th 319	6th 289	7th 245
TNB	10.85 ± 2.54 ^a^	11.31 ± 2.64 ^a^	11.72 ± 2.42 ^a^	12.07 ± 2.56 ^a^	11.91 ± 2.29 ^ab^	12.18 ± 2.29 ^bc^	12.16 ± 2.38 ^c^
NBA	9.34 ± 2.53 ^a^	10.19 ± 2.37 ^a^	10.36 ± 2.07 ^a^	10.39 ± 2.18 ^a^	10.32 ± 1.77 ^a^	10.45 ± 1.8 ^a^	10.38 ± 1.9 ^b^
NHOP	9.24 ± 2.53 ^a^	10.18 ± 2.36 ^a^	10.36 ± 2.07 ^a^	10.35 ± 2.17 ^a^	10.29 ± 1.76 ^a^	10.45 ± 1.8 ^a^	10.34 ± 1.9 ^b^
WLS	0.1 ± 0.36 ^a^	0.01 ± 0.12 ^b^	0 ± 0 ^b^	0.03 ± 0.25 ^b^	0.02 ± 0.2 ^b^	0 ± 0b	0.04 ± 0.25 ^b^
NS	1.11 ± 1.68 ^a^	0.8 ± 1.08 ^a^	1 ± 1.19 ^a^	1.24 ± 1.13 ^a^	1.13 ± 1.16 ^a^	1.2 ± 1.09 ^ab^	1.19 ± 1.34 ^b^
NM	0.6 ± 0.71 ^a^	0.4 ± 0.74 ^a^	0.38 ± 0.76 ^ab^	0.47 ± 0.65 ^abc^	0.45 ± 0.7 ^abc^	0.55 ± 0.84 ^bc^	0.61 ± 0.81 ^c^

Note: ^1^ TNB: total number born; NBA: number born alive; NHOP: number of healthy offspring produced; WLS: weak litter size; NS: number of stillbirths; NM: number of mummies. Different letters in the same row indicate significant differences (*p* < 0.05), while the same letters indicate no significant differences (*p* > 0.05). ^2^ Litters: litter size.

**Table 5:** Estimation of genetic parameters for six reproductive traits.

Traits ^1^	^2^ $\sigma_{a}^{2}$	^3^ $\sigma_{pe}^{2}$	^4^ $\sigma_{e}^{2}$	h2 (SE) ^5^
TNB	0.5457	0.2733	5.2795	0.0894 (0.0256)
NBA	0.4866	0.1206	4.6074	0.0918 (0.0240)
NHOP	0.4755	0.1183	4.5939	0.0895 (0.0237)
WLS	0.0003	<0.01	0.0402	0.0085 (0.0082)
NS	0.0046	0.0883	1.5071	0.0029 (0.0068)
NM	0.0073	< 0.01	0.5295	0.0136 (0.0102)

Note: ^1^ TNB: total number born; NBA: number born alive; NHOP: number of healthy offspring produced; WLS: weak litter size; NS: number of stillbirths; NM: number of mummies. ^2^ $\sigma_{a}^{2}$: additive genetic variance. ^3^ $\sigma_{pe}^{2}$: permanent environmental effect variance. ^4^ $\sigma_{e}^{2}:$: residual effect variance. ^5^ h2(SE): heritability (standard error).

**Table 6:** Polytropic SNPs in Jinwu pig.

SSC ^1^	Related Trait ^2^	SNP ^3^	Position	Corresponding *p*_Wald	Candidate Gene
18	TNB/NBA/NHOP	chr18_49194220 (rs326174997)	49,194,220	9.57 × 10^−9^ 2.42 × 10^−7^/3.05 × 10^−7^	*TNS3*
18	TNB/NBA/NHOP	chr18_48701969 (rs81233849)	48,701,969	3.56 × 10^−7^ 1.25 × 10^−7^/1.76 × 10^−7^	*Vopp1/PGAM2*
1	NBA/NHOP	chr1_172136167 (rs80793150)	172,136,167	4.28 × 10^−8^/4.53 × 10^−8^	*LRFN5*
6	NBA/NHOP	chr6_160159371 (rs332416322)	160,159,371	5.17 × 10^−7^ 7.10 × 10^−8^	*TUT4/ORC1/CC2D1B/ZFYVE9*

Note: ^1^ SSC: Sus scrofa chromosome; ^2^ TNB: total number born; NBA: number born alive; NHOP: number of healthy offspring produced; ^3^ SNP: single-nucleotide polymorphism.

## Data Availability

The original contributions presented in this study are included in the article/Supplementary Material. Further inquiries can be directed to the corresponding author.

## Supplementary Materials

The following supporting information can be downloaded at https://www.mdpi.com/article/10.3390/genes16050550/s1: Figure S1: Q-Q plots for six reproductive traits in Jinwu pig population. Table S1: Significant SNPs for six reproductive traits identified by GWAS (*p* < 1 × 10^−6^). Table S2: Genes with suggestive significant SNPs within 0.5 Mb of reproductive traits identified by GWAS. Table S3: Distribution of candidate genotypes and their relative frequencies.

## Abbreviations

The following abbreviations are used in this manuscript:

AI-REML	Average Information Restricted Maximum Likelihood
GS	Genomic selection
GWAS	Genome-wide association study
JW	Jinwu pig
MAS	Marker-assisted selection
NBA	Number of live births
NHOP	Number of healthy births
NJ	Neighbor-joining
NM	Number of mummified fetuses
NS	Number of stillbirths
PCA	Principal component analysis
PIC	Polymorphism information content
QTLs	Quantitative trait loci
SNP	Single-nucleotide polymorphism
TNB	Total number of births
WLS	Number of weak births
