# Supplementary material for: Statistical Analysis of Reproductive Traits in Jinwu Pig and Identification of Genome-Wide Association Loci
Source: Genes (Basel). 2025 Apr 30;16(5):550. doi: 10.3390/genes16050550 (PMC12110849; doi:10.3390/genes16050550)
Supplement: Supplementary file 1 [file genes-16-00550-s001.zip › Figure.S1.pdf]

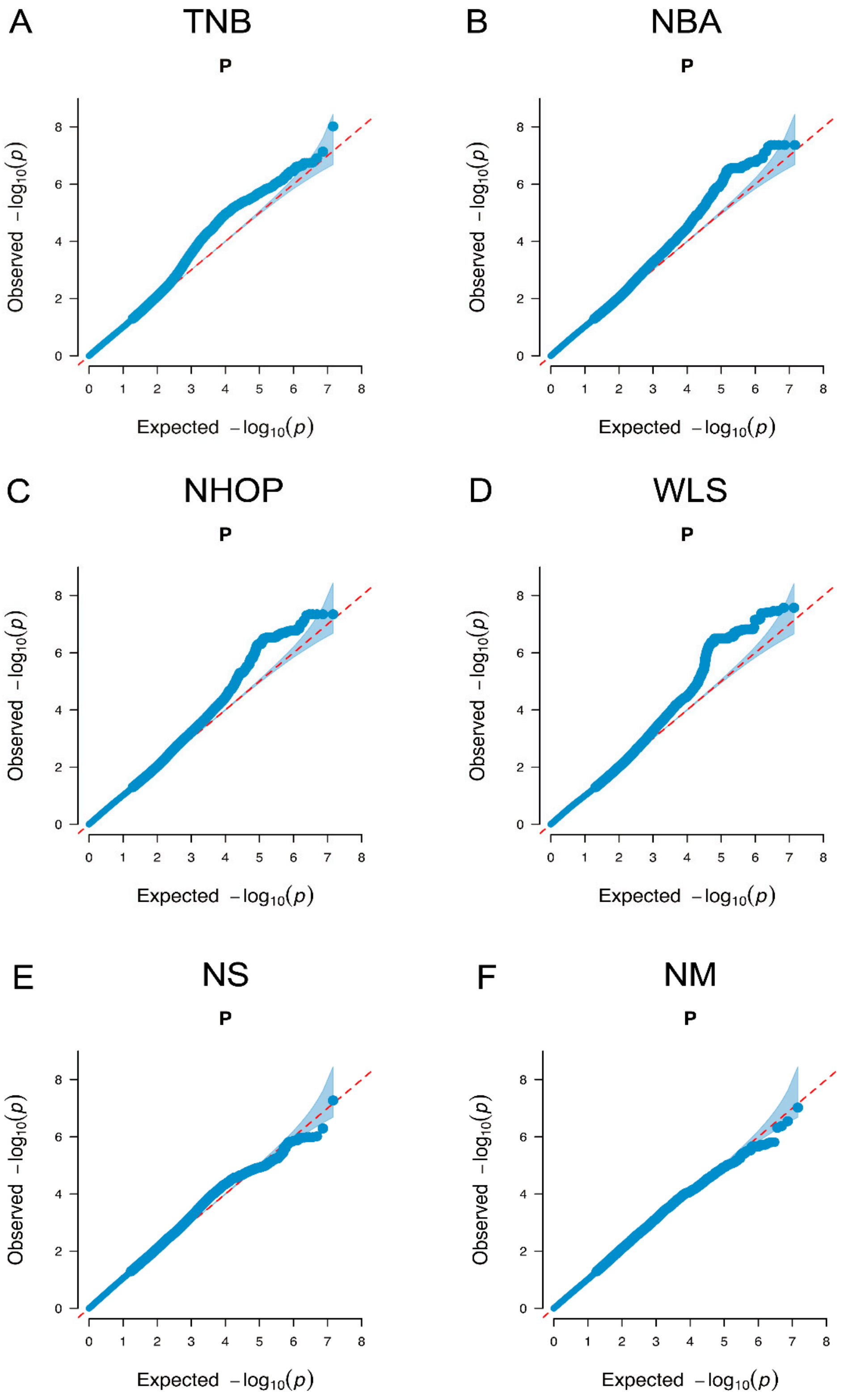


**Figure S1**. Q-Q plots of observed p-values for TNB, NBA, NHOP, WLS, NS, and NM traits. The y-axis represents the observed −log10-transformed p-values, and the x-axis represents the expected −log10-transformed p-values under the null hypothesis.
