# Supplementary material for: Statistical Analysis of Reproductive Traits in Jinwu Pig and Identification of Genome-Wide Association Loci
Source: Genes (Basel). 2025 Apr 30;16(5):550. doi: 10.3390/genes16050550 (PMC12110849; doi:10.3390/genes16050550)
Supplement: Supplementary file 1 [file genes-16-00550-s001.zip › Table.S3.pdf]

Table S3. Distribution of candidate genotypes and their relative frequencies

SNP	variety	TT	TC	CC
chr18_49194220	D	0.30	7.46	92.24
	JH	28.57	47.83	23.60
	JW	6.58	29.79	63.64
chr18_48701969	variety	GG	TG	TT
	D	81.49	15.22	3.28
	JH	4.97	22.36	72.67
	JW	24.18	50.48	25.34
chr1_172136167	variety	TT	TC	CC
	D	5.37	31.04	63.58
	JH	9.32	28.57	62.11
	JW	5.61	47.58	46.81
chr6_160159371	variety	AA	AG	GG
	D	1.19	18.51	80.30
	JH	13.66	44.10	42.24
	JW	0.39	12.57	87.04
